# *Mycobacterium avium* MAV2054 protein induces macrophage apoptosis by targeting mitochondria and reduces intracellular bacterial growth

**DOI:** 10.1038/srep37804

**Published:** 2016-11-30

**Authors:** Kang-In Lee, Jake Whang, Han-Gyu Choi, Yeo-Jin Son, Haet Sal Jeon, Yong Woo Back, Hye-Soo Park, Seungwha Paik, Jeong-Kyu Park, Chul Hee Choi, Hwa-Jung Kim

**Affiliations:** 1 Department of Microbiology, College of Medicine, Chungnam National University, Daejeon 35015, Republic of Korea; 2 Department of Medical Science, College of Medicine, Chungnam National University, Daejeon 35015, Republic of Korea

## Abstract

*Mycobacterium avium* complex induces macrophage apoptosis. However, the *M. avium* components that inhibit or trigger apoptosis and their regulating mechanisms remain unclear. We recently identified the immunodominant MAV2054 protein by fractionating *M. avium* culture filtrate protein by multistep chromatography; this protein showed strong immuno-reactivity in *M. avium* complex pulmonary disease and in patients with tuberculosis. Here, we investigated the biological effects of MAV2054 on murine macrophages. Recombinant MAV2054 induced caspase-dependent macrophage apoptosis. Enhanced reactive oxygen species production and JNK activation were essential for MAV2054-mediated apoptosis and MAV2054-induced interleukin-6, tumour necrosis factor, and monocyte chemoattractant protein-1 production. MAV2054 was targeted to the mitochondrial compartment of macrophages treated with MAV2054 and infected with *M. avium*. Dissipation of the mitochondrial transmembrane potential (ΔΨ_m_) and depletion of cytochrome *c* also occurred in MAV2054-treated macrophages. Apoptotic response, reactive oxygen species production, and ΔΨ_m_ collapse were significantly increased in bone marrow-derived macrophages infected with *Mycobacterium smegmatis* expressing MAV2054, compared to that in *M. smegmatis* control. Furthermore, MAV2054 expression suppressed intracellular growth of *M. smegmatis* and increased the survival rate of *M. smegmatis*-infected mice. Thus, MAV2054 induces apoptosis via a mitochondrial pathway in macrophages, which may be an innate cellular response to limit intracellular *M. avium* multiplication.

*Mycobacterium avium* complex (MAC), a member of the nontuberculous mycobacterium (NTM) group, is an opportunistic pathogen in humans and animals^1^ and is the most common cause of NTM lung disease worldwide^2^. MAC causes severe pulmonary and disseminated disease in immunocompromised hosts, particularly in individuals with AIDS^3^,^4^. MAC can also cause lung disease in apparently immunocompetent patients without underlying lung disease^5^. Although the diagnoses of disease due to NTM has increased worldwide in recent years^6^, little is known about the pathogenesis and virulent mechanism of NTM including MAC, particularly the detailed interactions between host cells and the bacteria.

Mycobacteria are intracellular facultative pathogens that grow within macrophages, which constitute the first line of innate immunity against most mycobacteria^7^,^8^. Therefore, mycobacterial virulence depends on the ability of these bacteria to invade, persist, and replicate within the hostile environment of macrophages. Apoptosis of macrophages infected with mycobacteria occurs during the disease course, and most studies have indicated that this is an innate cellular response to limit the multiplication of intracellular pathogens^9^,^10^. Most reports have indicated that apoptosis induction by *Mycobacterium tuberculosis* is inversely proportional to bacterial virulence^11^,^12^. MAC-infected macrophages also undergo apoptosis through p38 mitogen-activated protein kinase (MAPK), apoptosis signal-regulating kinase 1 (ASK1), and mitochondrial death signalling^13^ or tumour necrosis factor (TNF) and Fas^14^. Apoptosis of *M. avium*-infected macrophages limit bacterial viability, suggesting that apoptosis acts as a defence mechanism against this pathogen^15^. In contrast, another report indicated that MAC can survive in apoptotic macrophages and that many of the bacteria escape the dying cells and infect adjacent macrophages^16^. The exact role of macrophage apoptosis in MAC infection remains unclear.

Programmed cell death is emerging as a major effect of bacterial pathogenesis. Virulent *M. tuberculosis* doses inhibit host cell apoptosis, while also inducing pro-apoptotic signals. To improve the understanding of the cellular mechanisms of mycobacterial pathogenesis, it is important to identify and characterise the bacterial components involved in modulating macrophage apoptosis. Several apoptosis-inducing factors of *M. tuberculosis* have been identified: 19-kDa protein^12^,^17^ and lipoarabinomannan^18^ via Toll-like receptor 2, PE_PGRS33 with TNF-α-inducing ability^19^, 38-kDa protein with up-regulation of cell death receptor^20^, and heparin-binding hemagglutinin (HBHA) via mitochondrial targeting^9^. The sonic extracts from *M. avium* cause apoptosis of monocytes and macrophages^21^, indicating that MAC also contains a component involved in modulating apoptosis. Recently, we identified *M. avium* MAV2052 protein, which induced apoptosis in murine macrophages^22^. However, the MAC components involved in inhibiting or triggering apoptosis are not well-understood.

To identify valuable antigens in the complex mycobacterial antigen mixture, we fractionated the mycobacterial culture filtrate proteins by multistep chromatography and determined the immunoreactivty of each fraction^23^,^24^,^25^. Recently, we identified MAV2054 protein with strong immuno-reactivity in MAC pulmonary disease as well as in patients with tuberculosis by fractionation of *M. avium* culture filtrate proteins^26^. At the amino acid level, MAV2054 protein shares 100% homology with the 35-kDa major membrane protein 1 of *M. avium* subsp*. paratuberculosis. Mycobacterium leprae* major membrane protein, an immunodominant protein^27^, shows 92% identity with MAV2054. In this study, we investigated the biological effects of MAV2054 on macrophages. MAV2054 induced significant apoptosis in macrophages through the production of reactive oxygen species (ROS), loss of mitochondrial membrane potential, and the release of cytochrome *c*, and MAV2054 was efficiently targeted to the mitochondria of macrophages. Furthermore, *M. smegmatis* expressing MAV2054 increased macrophage apoptosis *in vivo* and *in vitro*, its growth within macrophages was suppressed, and MAV2054 expression attenuated the virulence of *M. smegmatis*.

## Results

### MAV2054 induces caspase-dependent macrophage apoptosis

**Figure 1:**
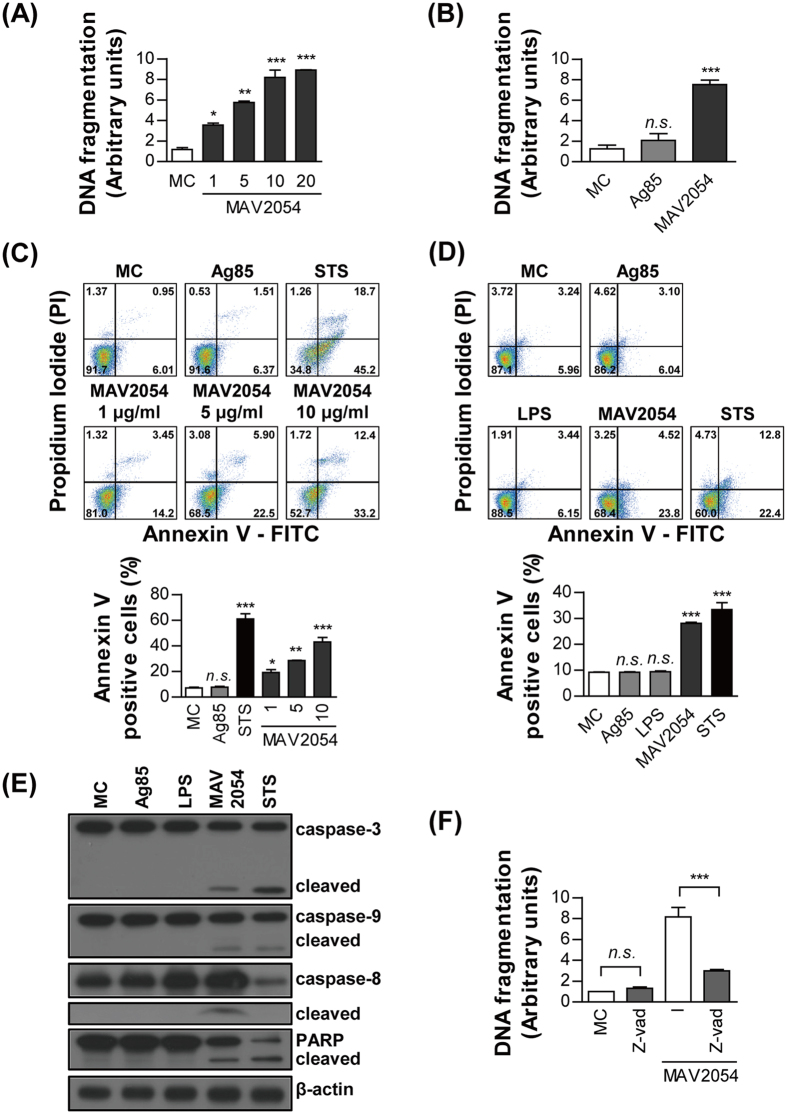
MAV2054 induces caspase-dependent macrophage apoptosis. (**A,B**) DNA fragmentation of RAW264.7 cells incubated with the indicated concentrations of MAV2054 or Ag85 for 24 h was measured by Cell Death Detection ELISA. Values represent the mean OD_405_ ± SD of at least three experiments. (**C,D**) Apoptosis of RAW264.7 cells treated with MAV2054 (1, 5, 10 μg/mL), Ag85 (10 μg/mL), or STS (50 nM) for 24 h was measured by flow cytometry using Annexin V/PI staining. Flow cytometric histograms are representative mean ± SD of three independent experiments (**E**) Total lysate from RAW264.7 cells exposed to each antigen for 24 h were immunoblotted against caspase 3, caspase 9, caspase 8, and PARP. To confirm equal protein loading, blots were re-probed with an antibody against β-actin. (**F**) RAW264.7 cells were incubated with MAV2054 (10 μg/mL) in the presence or absence of zVAD-fmk (50 μM). DNA fragmentation was measured as described above. Results are the mean ± SD of three independent experiments. *P < 0.05, **P < 0.01, and ***P < 0.001 for antigen treatments compared to untreated cells (medium control, MC).

Since *M. avium* MAV2054 is a major membrane protein and an immunodominant antigen^26^,^27^, we first determined whether MAV2054 could induce macrophage activation. Incubation with MAV2054 converted RAW264.7 cells into differentiated macrophage-like cells at an early stage, but cell death patterns morphologically appeared after 24 h. We tested whether MAV2054 induced apoptosis using a cell death ELISA kit to assess DNA fragmentation. MAV2054 induced significant apoptosis in a dose-dependent manner in RAW264.7 cells (Fig. 1A). We used native antigen 85 complex (Ag85) as an unrelated mycobacterial control protein. Ag85 is the major secreted and fibronectin-binding protein of *M. tuberculosis,* and shows strong immunoreactivity^28^,^29^. Apoptosis was significantly greater in cells treated with MAV2054 as compared to untreated control or cells treated with Ag85 (Fig. 1B). In addition, based on the results from MTT assay, it appeared that MAV2054 induced cell death in a dose-dependent manner but not antigen 85 complex (Ag85) or LPS (Supplementary Figure 1a). MAV2054-induced cell death did not occur because of necrosis because lactate dehydrogenase (LDH) was not detected in the culture supernatant of cells treated with MAV2054 (Supplementary Figure 1b). Flow cytometry results revealed that the population of Annexin-V-positive cells in MAV2054-treated RAW264.7 cells was significantly increased in a dose-dependent manner (Fig. 1C). Staurosporine (STS) was used as a positive control. Similar results were observed in bone marrow-derived macrophages (BMDMs) (Fig. 1D). However, neither LPS nor Ag85 induced significant apoptosis in BMDMs. We further assessed whether MAV2054 induced macrophage apoptosis in a caspase-dependent manner. As shown in Fig. 1E, cleavage of caspase-3, caspase-9, and poly ADP ribose polymerase (PARP) in cells treated with MAV2054 for 24 h was observed and was comparable to that in STS-treated cells. In addition, MAV2054 induced the activation of caspase-8. Inhibition of caspases by a pan-caspase inhibitor, z-VAD-fmk, attenuated MAV2054-induced DNA fragmentation (Fig. 1F). Overall, these data support that MAV2054 caused caspase-dependent apoptosis in macrophages.

### MAV2054 induces production of proinflammatory cytokines and chemokines

**Figure 2:**
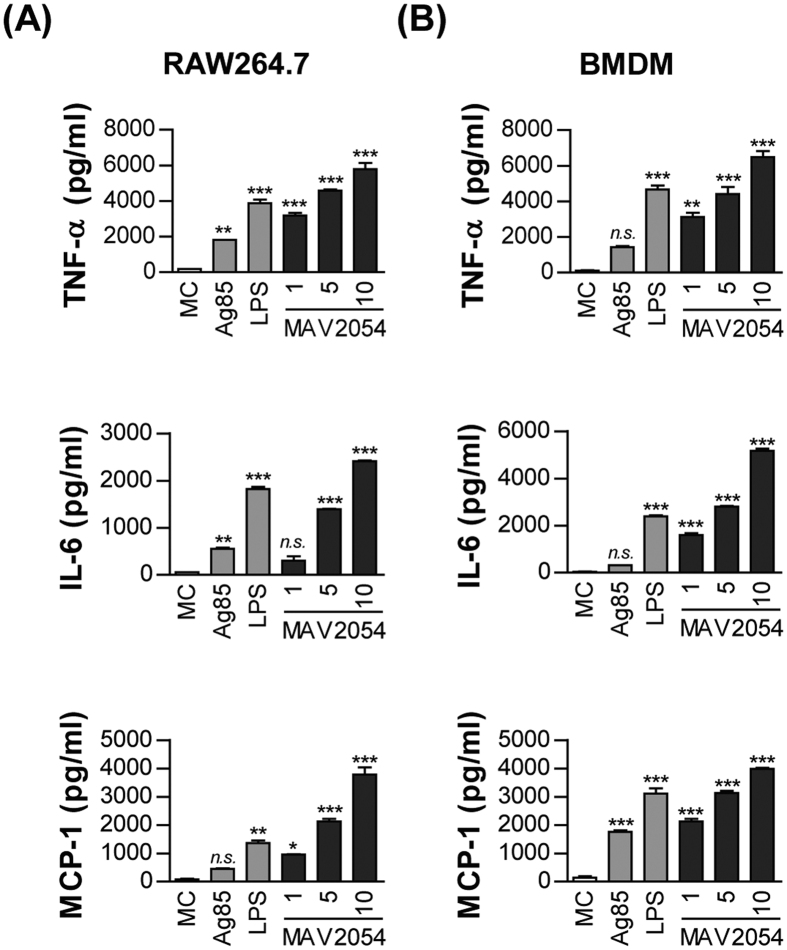
Production of TNF-α, IL-6, and MCP-1 from RAW264.7 cells or BMDMs stimulated with MAV2054 protein. RAW264.7 cells **(A**) and BMDMs (**B**) were stimulated with MAV2054 (1, 5, 10 μg/mL), Ag85 (10 μg/mL), and LPS (100 ng/mL) for 24 h. TNF-α, IL-6 and MCP-1 levels in the culture supernatants were measured by ELISA. The results are the mean ± SD from three experiments. *P < 0.05, **P < 0.01, and ***P < 0.001 for antigen treatments compared to untreated medium control (MC).

TNF-α and IL-6 are pro-inflammatory cytokines that participate in macrophage activation and regulate the cell death pathway during infection^30^,^31^,^32^. We tested whether MAV2054 could stimulate a macrophage to secrete these cytokines. RAW264.7 cells or BMDMs treated with MAV2054 produced significant TNF-α, IL-6, and MCP-1 compared to untreated controls or Ag85 (Fig. 2). Although endotoxin content was continuously monitored during preparation of the recombinant protein, we further assessed LPS contamination by treatment with proteinase K or heat denaturation, which abrogated the ability of MAV2054 to stimulate macrophages (Supplementary Figure 2). Polymyxin B treatment did not affect MAV2054 function, whereas the effects of LPS were significantly inhibited by polymyxin B.

### ROS are required for apoptosis and pro-inflammatory cytokine production induced by MAV2054

**Figure 3:**
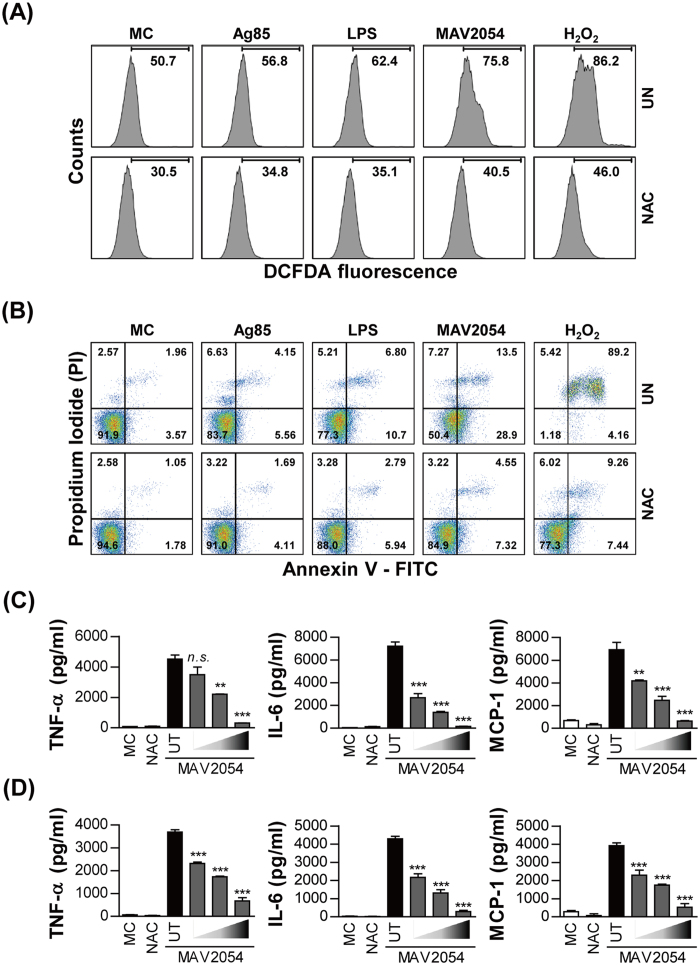
MAV2054-induced apoptosis and cytokine production are mediated by ROS production. (**A,B**) RAW264.7 cells were treated with MAV2054 (10 μg/mL), Ag85 (10 μg/mL), LPS (100 ng/mL), or H_2_O_2_ (500 μM) in the presence or absence of NAC (10 mM) for 24 h. ROS levels (**A**) were measured by flow cytometry after DCFDA (10 μm) treatment. Data are representative flow cytometric analysis from three independent experiments. Apoptosis of RAW264.7 cells (**B**) was measured by flow cytometry using Annexin V/PI staining. Flow cytometric data shown are representative of at least three experiments. (**C**) RAW264.7 cells or (**D**) BMDMs pre-treated with or without antioxidant NAC (10, 20, 30 mM) for 1 h were stimulated with MAV2054 (10 μg/mL) for 24 h. The levels of TNF-α, IL-6, and MCP-1 from the culture supernatants were measured using by ELISA. All values are mean ± SD of three experiments. **P < 0.01 and ***P < 0.001 for NAC treatments compared to MAV2054 treatment alone. MC, medium control.

It is well-known that ROS can induce apoptosis and pro-inflammatory cytokine production in macrophages^33^,^34^. To examine the involvement of ROS generation on the effects of MAV2054 in RAW264.7 cells, the oxidation of 2′,7′-dichlorodihydrofluorescein diacetate (H2DCFDA) was assessed by flow cytometry. MAV2054 induced a significant increase in intracellular ROS in macrophages compared to Ag85 or medium control (Fig. 3A). ROS production in MAV2054-treated RAW264.7 cells was increased in a dose-dependent manner (Supplementary Figure 3). MAV2054-mediated ROS production was dramatically reduced by pre-treatment with the ROS scavenger *N*-acetyl-L-cysteine (NAC). Next, we examined the apoptotic response in the presence of NAC to investigate the interplay between apoptosis and ROS generation. Pre-treatment with NAC significantly inhibited Annexin V-fluorescein isothiocyanate (FITC)-positive cells induced by MAV2054 in RAW264.7 cells (Fig. 3B). These results suggest that increased ROS is essential for the apoptotic response caused by MAV2054. In addition, MAV2054-induced TNF-α, IL-6, and MCP-1 levels were also significantly decreased in RAW264.7 cells or BMDMs by pre-treatment with NAC in a dose-dependent manner (Fig. 3C and D), indicating that ROS, which are induced by MAV2054, are associated with the production of these cytokines and chemokines.

### JNK activation is required for apoptosis induced by MAV2054

**Figure 4:**
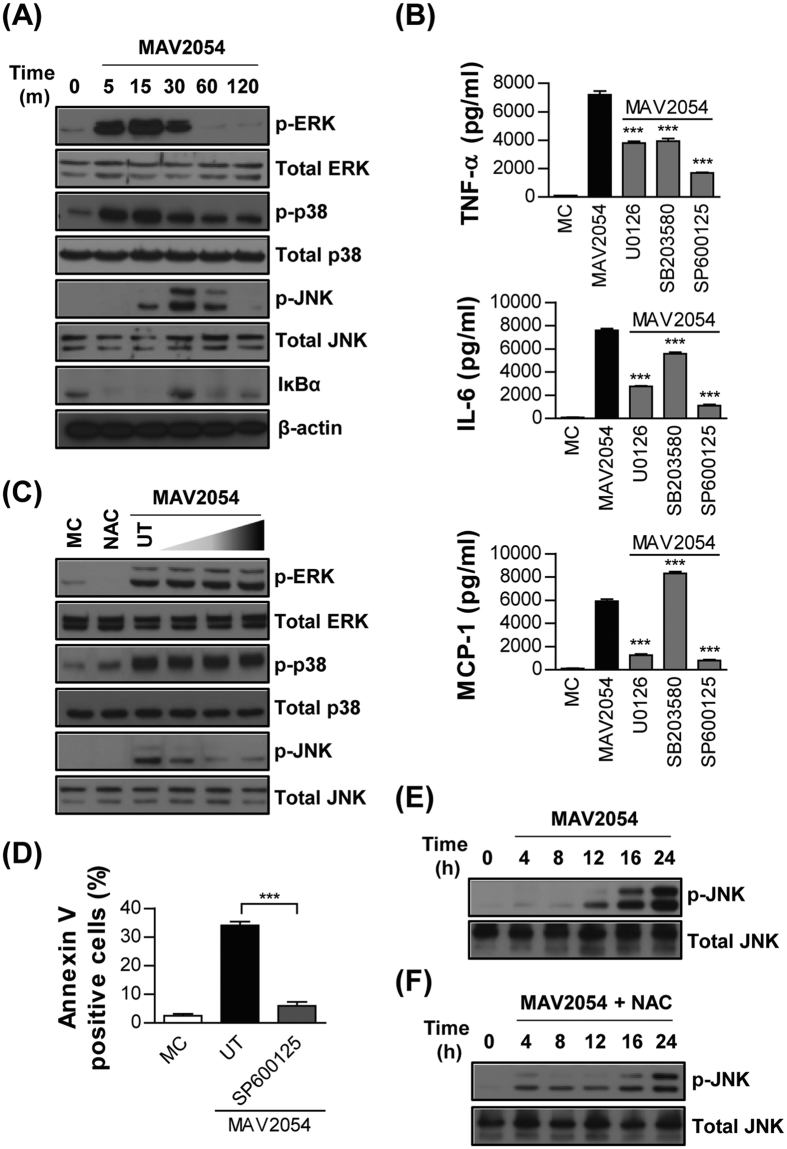
MAV2054-induced JNK activation is associated with ROS production and apoptosis. (**A**) RAW264.7 cells stimulated with MAV2054 for the indicated times were lysed and the proteins in the total cell lysate were separated by SDS–PAGE, followed by immunoblotting with antibodies against phospho-ERK1/2, ERK1/2, phospho-p38, p38, phospho-JNK, JNK, IκBα, and β-actin. This image is representative of three experiments showing similar results. (**B**) BMDMs pre-incubated with inhibitors of ERK (U0126), p38 (SB203580), JNK (SP600125), and NF-ĸB (BAY11-7082) for 1 h were treated with MAV2054 (10 μg/mL) for 24 h. The levels of TNF-α, IL-6, and MCP-1 were measured by ELISA. ***P < 0.001 for inhibitor treatments compared to MAV2054 treatment alone. (**C**) RAW264.7 cells pre-incubated with or without NAC (10, 20, 30 mM) for 1 h were stimulated with MAV2054 (10 μg/mL) for 30 min. Activity of MAPKs was determined by immunoblot analysis. The data are representative of three independent experiments with similar results. (**D**) RAW264.7 cells were incubated with JNK inhibitor (SP600125, 10 μM) for 1 h prior to treatment with MAV2054 (10 μg/mL). After 24 h, apoptosis was analysed by flow cytometry. (**E,F**) JNK activity of MAV2054 (10 μg/mL)-stimulated RAW264.7 cells without (**E**) or with pre-treatment of NAC (**F**) up to 24 h was determined by immunoblot analysis. All values are the mean ± SD of three experiments. MC, medium control; UT, untreated.

MAPKs are signalling factors known to play a critical role in inducing inflammatory cytokine production^35^ and have been linked to *M. avium*-induced macrophage apoptosis^13^. Thus, we examined whether MAPKs was activated in response to macrophage stimulation with MAV2054. The phosphorylation profiles of extracellular receptor kinase 1/2 (ERK1/2), p38, JNK, and IκBα in RAW264.7 cells were analysed at various time-points after stimulation with MAV2054. ERK1/2, p38, and JNK were strongly phosphorylated at 15–30 min after stimulation (Fig. 4A). In addition, the expression of IκBα was rapidly lost at 5–15 min and then reappeared. These results suggest that MAV2054-induced macrophage activation is mediated by signalling pathways involving MAPKs and nuclear factor (NF)-κB. To confirm the role of MAPKs and NF-κB in MAV2054-induced cytokine production, BMDMs were pre-treated with a specific inhibitor of ERK1/2 (U0126), p38 (SB203580), JNK (SP600125), or NF-κB (BAY 11–7082) for 1 h, and cytokine levels were assayed at 24 h after MAV2054 stimulation. TNF-α, IL-6, and MCP-1 were significantly suppressed by all inhibitors in a dose-dependent manner, except for MCP-1 which was significantly increased by the p38 inhibitor (Fig. 4B).

Next, we assessed whether NAC could block the phosphorylation of MAPKs induced by MAV2054. As shown in Fig. 4C, NAC pre-treatment significantly inhibited JNK activation in a dose-dependent manner. However, the phosphorylation of ERK1/2 and p38 was not suppressed in the presence of NAC. These data suggest that ROS are involved in MAV2054-mediated JNK activation. We further confirmed the involvement of JNK in MAV2054-mediated apoptosis. Flow cytometry showed that the JNK inhibitor significantly suppressed MAV2054-induced macrophage apoptosis (Fig. 4D). However, the p38 inhibitor could not suppress MAV2054-mediated Annexin V-FITC-positivity in cells (Supplementary Figure 4).

In addition, we examined the status of JNK phosphorylation after MAV2054 treatment. As shown in Fig. 4E, phosphorylation of JNK was significantly increased in a time-dependent manner and persisted for the duration of the experiment. To further analyse whether MAV2054-induced ROS generation was responsible for JNK activation, cells were pre-treated with NAC and then exposed to MAV2054 for up to 24 h. Pre-treatment with NAC suppressed the phosphorylation of JNK caused by MAV2054 (Fig. 4F). Overall, our results suggest that ROS/JNK signalling is involved in MAV2054-mediated apoptosis in macrophages.

### MAV2054 decreases mitochondrial transmembrane potential (ΔΨ_m_) and increases cytochrome *c* release

**Figure 5:**
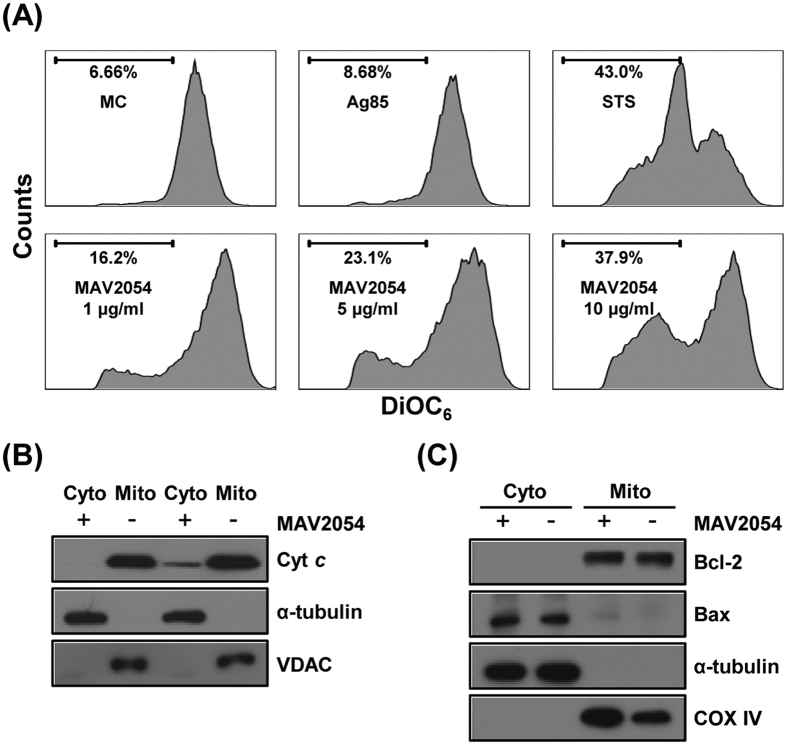
MAV2054 induces ΔΨ_m_ collapse and cytochrome *c* release. RAW264.7 cells treated with Ag85 (10 μg/mL) or MAV2054 (1, 5, 10 μg/mL) for 24 h. (**A**) The cells were stained with DiOC_6_ (10 nM). The fluorescence activity of data are representative flow cytometric analysis from three independent experiments. (**B,C**) Mitochondrial and cytosolic fractions were prepared, and aliquots containing 20 μg of protein were subjected to western blot analysis and probed with antibodies against cytochrome *c*, Bcl-2 or Bax. α-Tubulin and VDAC or COX IV were used as markers for the cytosolic and mitochondrial fractions, respectively. The results are representative of three independent experiments.

The mitochondria play a central role in the response to apoptotic stimuli, allowing signals from various inputs to converge^36^. Damage to the mitochondrial membrane results in release the several death-promoting factors such as cytochrome *c*. We tested whether MAV2054 treatment affected the structural and biochemical integrity of the mitochondria. Mitochondrial damage was assessed by examining ΔΨ_m_, which was determined by staining cells with 3,3′-dihexyloxacarbocyanine (DiOC_6_) for flow cytometric analysis. As shown in Fig. 5A, a significant dose-dependent decrease in ΔΨ_m_ was observed in RAW264.7 cells incubated with MAV2054, as indicated by the decrease in DiOC_6_ intensity compared to that in untreated or Ag85-treated cells. Next, we tested whether Bax, a pro-apoptotic Bcl-2 family member, was translocated to the mitochondria, and cytochrome *c* was released into the cytoplasm from the mitochondria. An increase in cytochrome *c* in the cytosolic fraction was detected in cells treated with MAV2054 following subcellular fractionation and western blot analysis (Fig. 5B). However, MAV2054 did not cause Bax translocation into the mitochondria (Fig. 5C). These findings suggest that the apoptotic effect of MAV2054 on macrophages is associated with mitochondrial damage and cytochrome *c* release.

### MAV2054 is targeted to the mitochondria

**Figure 6:**
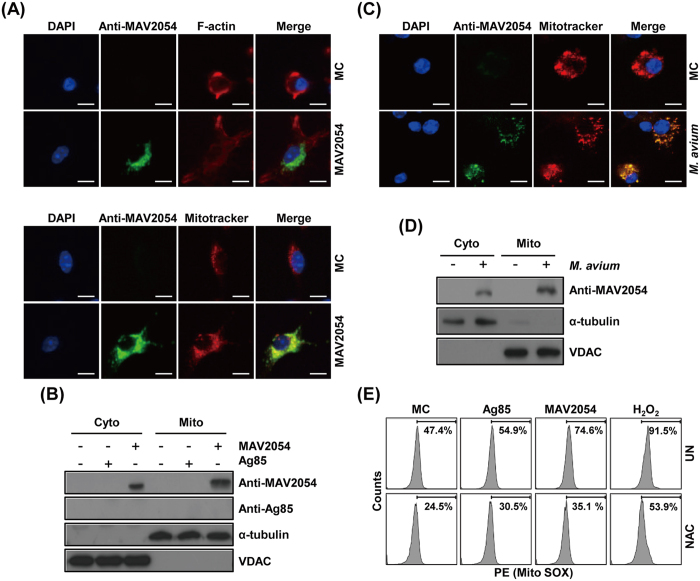
Subcellular localization of MAV2054 in macrophages. BMDMs were treated with MAV2054 (10 μg/mL) or Ag85 (10 μg/mL) for 24 h or infected with *M. avium* at MOI of 10 for 20 h. (**A**) Representative confocal images of medium control (MC) and MAV2054-treated cells. DNA visualized with DAPI (blue), MAV2054 (green), and F-actin (red) in the upper panel, and MAV2054 (green) and Mitotracker (Red) in lower panel. Yellow in merged images indicates co-localization. Scale bar, 10 μm. (**B**) Mitochondrial and cytosolic fractions were separated from cell lysates and examined by western blotting using specific antibodies against MAV2054, Ag85, VDAC, and α-tubulin. (**C**) Representative confocal images of MC and *M. avium* infected cells. Scale bar, 10 μm. (**D**) Localization of MAV2054 in BMDMs infected with *M. avium* at MOI of 10 for 4 h was analysed by western blotting using specific antibodies against MAV2054, VDAC, and α-tubulin. (**E**) RAW264.7 cells were treated with MAV2054 (10 μg/mL) or Ag85 (10 μg/mL), and H_2_O_2_ (500 μm) for 24 h. Cells were labelled with MitoSOX for 30 min and analysed for mitochondrial ROS levels by flow cytometry. A shift in the cell population to the right indicates the amount of ROS generation. The data are representative of three independent experiments.

MAV2054-induced apoptosis occurred following the release of cytochrome *c* but was not related to Bcl-2 family signalling, indicating that cytochrome *c* release is due to factors other than MAV2054. It was previously demonstrated that a protein is targeted to the mitochondria and induces the release of cytochrome *c* to enhance the intrinsic response of apoptosis^17^. We also found that *M. tuberculosis* HBHA induces cytochrome *c* release through a direct interaction with the mitochondria^9^. Therefore, we evaluated where MAV2054 is localized in the mitochondria of cells treated with this protein. Confocal microscopy analysis revealed the presence of MAV2054 in the cytosol and mitochondria of MAV2054-treated cells, based on the significant overlap between MAV2054 and Mitotracker signals (Fig. 6A). Subcellular fractionation and western blot analysis consistently showed that large amounts of MAV2054 were detected in the cytosol and mitochondrial fractions, but the unrelated mycobacterial antigen Ag85 was not detected in the cytosol and mitochondrial fraction of BMDMs treated with Ag85 (Fig. 6B). Next, we further tested whether MAV2054 was transported to the mitochondria during *M. avium* infection. Confocal microscopy of infected cells revealed that some MAV2054 was colocalised with the mitochondria (Fig. 6C). The purified mitochondrial fraction and cytosolic fraction of these cells contained equal amounts of MAV2054 protein (Fig. 6D). In fact, the protein homologous to MAV2054 in *M. leprae* and *M. avium* subsp*. paratuberculosis* is a membrane protein^27^,^37^. To determine the location of MAV2054 in *M. avium*, the bacteria were fractionated into cytosol, cell wall, and cell membrane components (Supplementary Figure 5a). Based on the results of western blotting, it appeared that MAV2054 was detected in both, the cell wall and the membrane fractions, but not in the cytosol fraction (Supplementary Figure 5b). Therefore, we further confirmed whether MAV2054 was released from phagosomes containing *M. avium*. The infected bacteria located in the phagosome and MAV2054 was detected in the phagosome, cytosol, and mitochondria (Supplementary Figure 6). Taken together, these findings demonstrate that MAV2054 is efficiently localised to the mitochondria of macrophages infected with *M. avium*.

Mitochondrial ROS generation causes ΔΨ_m_ disruption and apoptotic cell death^38^,^39^. We determined whether treatment of macrophages with MAV2054 induced mitochondrial ROS generation. The MitoSOX dye selectively binds to superoxide in the mitochondria. Flow cytometry analysis revealed a significant shift to a high intensity of this dye in RAW264.7 cells treated with MAV2054 compared to in untreated controls or Ag85 (Fig. 6E). These results demonstrated that ROS production in macrophages treated with MAV2054 was mainly increased through the mitochondria.

To determine whether the apoptotic activity of MAV2054 protein was dependent on the putative mitochondrial localization sequence (MLS), we wanted to identify the presence of MLS in the MAV2054 protein using the MitoProt II v1.101 (https://ihg.gsf.de/ihg/mitoprot.html). It was predicted that the MLS was located in the N-terminal of the MAV2054 protein. Three truncated forms of the recombinant protein with or without these target sequences were purified (Supplementary Figure 7b), and their activity was analyzed. FACS and DNA fragmentation assay revealed that the apoptotic response in macrophages treated with the truncated form lacking the putative mitochondrial target sequences was decreased (Supplementary Figure 7c and 7d). In addition, ROS production, caspase activation, and the decreased ΔΨ_m_ of MAV2054 correlated with the mitochondrial target sequences (Supplementary Figure 7e to 7g).

### *Mycobacterium smegmatis* expressing MAV2054 increases apoptotic response of infected macrophages

**Figure 7:**
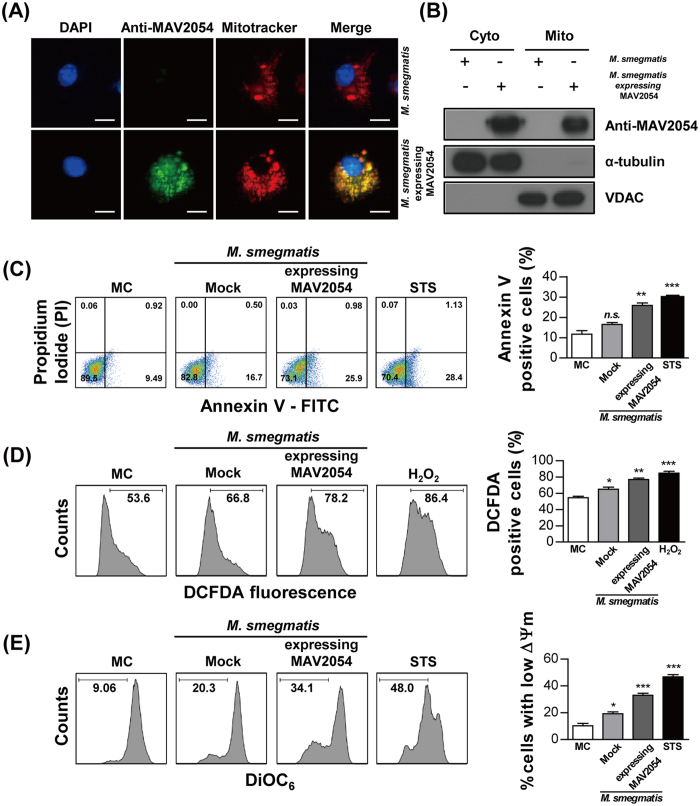
MAV2054 localization and increased apoptosis in macrophages infected with *M. smegmatis* expressing MAV2054. BMDMs were infected with *M. smegmatis* expressing MAV2054 or empty plasmid (mock) at an MOI of 5 for 20 h. (**A**) Representative confocal images of BMDM infected with mock strain and *M. smegmatis* expressing MAV2054. DNA visualized with DAPI (blue), MAV2054 (green), and Mitotracker (Red). Yellow in merged images indicates co-localization. Scale bar, 10 μm. (**B**) Mitochondrial and cytosolic fractions were separated from the cell lysates and examined by western blotting using specific antibodies against MAV2054, VDAC, and α-tubulin. (**C**) Apoptosis of the infected cells was analysed by flow cytometry using Annexin V/PI staining. (**D**) The infected cells were labelled with DCFDA (10 μm) for 30 min and analysed for ROS production. A shift in the cell population to the right indicates ROS generation. (**E**) The fluorescence activity of DiOC_6_ was determined by flow cytometry using DiOC_6_ (10 nM) staining. A shift in the cell population to the left indicates a loss of ΔΨ_m_. Flow cytometric histograms are representative mean ± SD of three independent experiments. *P < 0.05, **P < 0.01, ***P < 0.001 for infected cells compared to uninfected cells. MC, medium control.

To confirm the effects of MAV2054 during infection, *M. smegmatis* expressing MAV2054 was generated and the protein expression in this bacteria was confirmed by western blotting (Supplementary Figure 8). First, we confirmed whether MAV2054 was also localized to the mitochondria in BMDMs infected with *M. smegmatis* expressing MAV2054. Confocal microscopy and western blotting results revealed that some MAV2054 colocalised with the mitochondria (Fig. 7A and B). However, MAV2054 was not detected in cells infected with *M. smegmatis* expressing an empty plasmid (mock). Next, we analysed the effect of MAV2054 on macrophages in the context of the bacterium. A significant increase in the number of apoptotic cells and increased ROS production were observed in BMDMs infected with *M. smegmatis* expressing MAV2054 compared to in BMDMs infected with the *M. smegmatis* mock strain (Fig. 7C and D). Furthermore, more significant ΔΨ_m_ collapse was detected in cells infected with *M. smegmatis* expressing MAV2054 (Fig. 7E). These findings suggest that MAV2054 affects macrophage apoptosis during bacterial infection through mitochondrial targeting of this protein.

### MAV2054 expression increases the survival rate of mice infected with *M. smegmatis*

**Figure 8:**
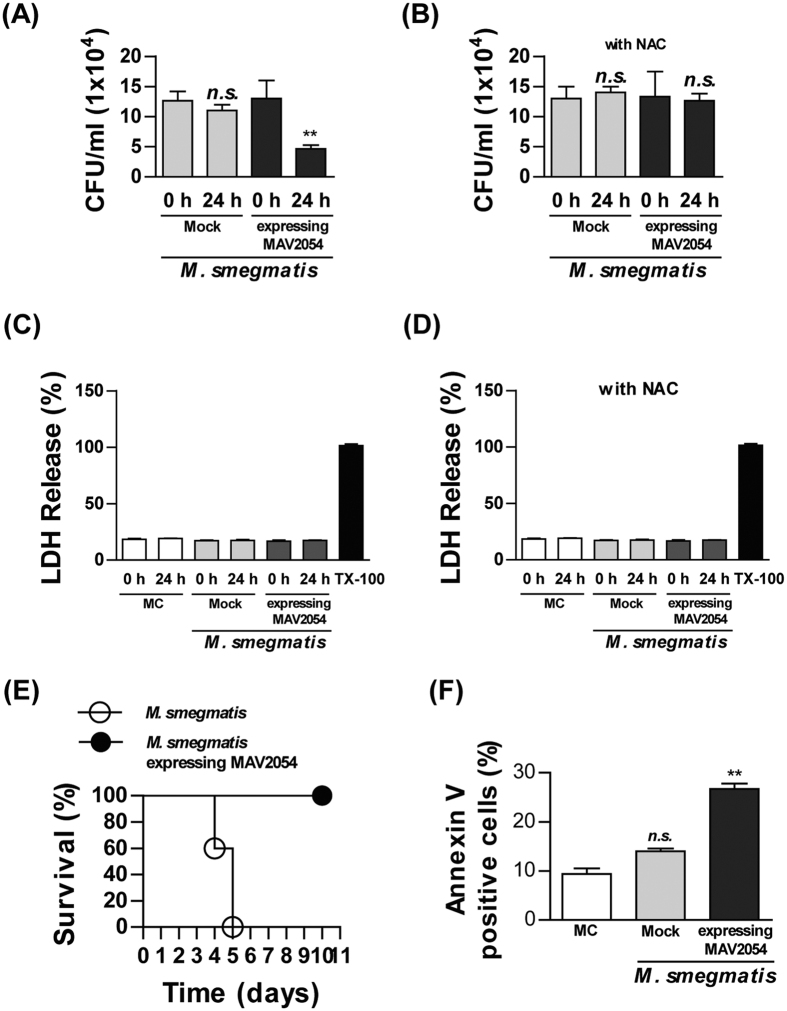
MAV2054 expression suppresses *M. smegmatis* intracellular growth and increases survival rate of mouse infected with *M. smegmatis.* BMDMs were infected with *M. smegmatis* expressing MAV2054 or *M. smegmatis* at an MOI of 5 for 24 h without (**A,C**) or with NAC (**B,D**). (**A,B**) At 0 h and 24, intracellular growth was determined by serial dilution of cell lysate and plating on 7H10 agar. **P < 0.01 for the comparison of infected cells with *M. smegmatis* expressing MAV2054 and *M. smegmatis*. (**C,D**) After 24 h, LDH release from the culture supernatants was measured using a Cytotoxicity Detection Kit. Positive controls were generated by treating cells with 1% Triton X-100 (TX-100) for 1 h prior to the assay. The results are the mean ± SD from three experiments. (**E**) Survival of C57BL/6 mice (*n* = 5 per group) infected with *M. smegmatis* expressing MAV2054 or *M. smegmatis* strain intravenously at a high dose (1 × 10^7^ CFU per mouse). Representative data from two experiments are shown. (**F**) Apoptotic population of alveolar macrophages from mouse (*n* = 3 per group) infected with the bacteria was analysed by flow cytometry using CD11b and F4/80, and Annexin V/PI staining at 3 days post-infection. The results are the mean ± SD of three mouse experiments. **P < 0.01 for infected mouse compared to non-infected mice.

It has been reported that more virulent strains of *M. tuberculosis* grow rapidly inside macrophages^40^, indicating that the intracellular survival rate of mycobacteria in macrophages can be used to indirectly assess their virulence. Therefore, to confirm the role of MAV2054-induced apoptosis, we measured the intracellular growth of *M. smegmatis* expressing MAV2054 in macrophages. Intracellular growth of *M. smegmatis* expressing MAV2054 at 24 h after infection was significantly decreased compared to the *M. smegmatis* mock strain (Fig. 8A). The reduced survival rate of *M. smegmatis* expressing MAV2054 was recovered by pre-treatment with NAC (Fig. 8B). In contrast, there was no significant difference in LDH release between cells infected with *M. smegmatis* and *M. smegmatis* expressing MAV2054 regardless of pre-treatment with NAC (Fig. 8C,D). Next, we confirmed the effect of MAV2054 expression in *M. smegmatis in vivo. Mycobacterium smegmatis* is generally considered a non-pathogenic organism. However, high-dose intravenous infection was fatal in C57BL/6 mice^41^. The survival rates of mice after intravenous *M. smegmatis* infection (1 × 10^7^ CFU) were determined. All mice infected with *M. smegmatis* expressing MAV2054 survived for >10 days, whereas all mice inoculated with the *M. smegmatis* strain died by 5 days post-infection (Fig. 8E). FACS analysis of alveolar macrophages collected at 3 days post-infection revealed that *M. smegmatis* expressing MAV2054 induced significant higher apoptosis of macrophages than in the mock strain. These results suggest that MAV2054-induced macrophage apoptosis is related to attenuation of *M. smegmatis* virulence.

## Discussion

MAC live within macrophages, and infection of macrophages by MAC can lead to their apoptotic death; the induction of macrophage apoptosis is strain-specific^13^,^14^,^15^,^42^. Several components of *M. tuberculosis* that are involved in triggering apoptosis and their inducing mechanisms have been determined. However, the MAC components that evoke apoptotic response have not been identified. MAV2054 is a major membrane protein that plays a role in the invasion of bovine epithelial cells in *M. avium* subsp. *paratuberculosis*^37^. In this study, we demonstrated that intracellular MAV2054 targets the mitochondria in macrophages, leading to ΔΨ_m_ dissipation, and eventual apoptosis. Our results show that MAC also contains a specific component that triggers host apoptotic responses via a mitochondria-dependent pathway during *M. avium* infection.

ROS molecules are involved in a variety of cellular signalling pathways, such as promoting the production of pro-inflammatory cytokines^43^,^44^. Increased intracellular ROS levels can trigger apoptosis by activating the mitochondrial-dependent cell death pathway^43^. Our data suggest that increased ROS is essential for MAV2054-mediated macrophage apoptosis and MAV2054-induced TNF-α, IL-6, and MCP-1 production. Other reports also showed that ROS are involved in *M. avium*- and its sonic extracts-induced macrophage apoptosis^13^,^21^. We also demonstrated that ROS production is required for macrophage apoptosis induced by *M. avium* MAV2052^22^.

It is well-known that mycobacteria induce apoptosis via extrinsic and intrinsic pathways or a caspase independent pathway. *M. avium*-induced apoptosis is mediated by TNF and Fas^14^, indicating that an extrinsic pathway is involved. The Fas-associated death domain-mediated caspase 8 activation through ASK1/p38 MAPK signalling, as well as caspase 9 activation via the intrinsic (mitochondrial) pathway, appear to be involved in the signalling of apoptosis induced by *M. avium*^13^. As expected, we also observed the activation of caspase-3, caspase-9, and subsequent cleavage of PARP after incubation of macrophages with MAV2054. Activation of JNK, but not p38, was involved in MAV2054-mediated apoptosis. In fact, *M. avium*-mediated ASK1/p38 activation is triggers subsequent Fas-associated, death domain-mediated caspase 8 activation, which is involved in the extrinsic pathway^13^. Although JNK and p38 were strongly phosphorylated in MAV2054-induced macrophages, p38 may not be required for MAV2054-mediated apoptosis because MAV2054 induced apoptosis via the mitochondrial pathway. Increasing evidence indicates a crucial role of JNK in mitochondria dysfunction and the subsequent initiation of apoptosis^45^. Particularly, it has been reported that various stimulants such as a natural derivative compound induce apoptosis with ROS-dependent JNK activation in target cells^46^,^47^,^48^. Our results additionally showed that NAC inhibited MAV2054-induced JNK activation, which was required for MAV2054-mediated apoptosis, suggesting that ROS/JNK signalling is involved in MAV2054-mediated apoptosis. In addition, MAV2054 induced activation of caspase 8, which results from ROS accumulation. ROS generation has been reported to activate ASK1, and increase the activity of caspase 8^49^,^50^.

Mitochondria are central organelles in which a variety of key events in intrinsic apoptosis occur, including the release of cytochrome *c*, changes in electron transport, and pro- and anti-apoptotic molecule activity^43^. Mitochondrial damage and cytochrome *c* release are considered to be critical causes of macrophage apoptosis mediated by *M. tuberculosis*^51^,^52^ or *M. avium*^14^. Recently, we demonstrated that the *M. tuberculosis* HBHA protein is targeted to the mitochondria of macrophages, which leads to apoptosis through ROS production, mitochondrial translocation of Bax, ΔΨ_m_ collapse, and cytochrome *c* release^9^. Here, we found that the *M. avium* MAV2054 protein was targeted to the mitochondrial compartment of macrophages treated with MAV2054 and infected with *M. avium*. Dissipation of ΔΨ_m_ and depletion of cytochrome *c* also occurred in the mitochondria of MAV2054-treated macrophages. Furthermore, MAV2054 was transported to the mitochondria of BMDMs infected with *M. smegmatis* expressing MAV2054. However, we found no evidence of mitochondrial translocation of Bax induced by MAV2054, indicating that molecules are not involved in MAV2054-induced cell death. ROS are predominantly produced in the mitochondria and lead to the modulation of ΔΨ_m_, which finally results in apoptosis^39^. Our data indicate that ROS generated in macrophages treated with MAV2054 were mainly produced in the mitochondria. Therefore, mitochondrial localization of MAV2054 may lead to ROS generation, subsequently affecting ΔΨ_m_ loss and cytochrome *c* release; these processes play an essential role in MAV2054-induced apoptosis. Another possibility is that MAV2054 induces mitochondrial dysfunction via ROS-dependent JNK activation, because significant evidence has shown that JNK activation leads to mitochondrial-dependent apoptosis^45^. However, we could not identify which host molecules physically and functionally interacted with intracellular MAV2054 or the molecular mechanism related to mitochondrial dysfunction. Several mitochondria-targeted proteins expressed by pathogens interact with voltage-dependent anion channels (VDACs)^53^,^54^. Recombinant MAV2054 used in this study was a His-tagged fusion protein. In fact, we tested whether MAV2054 interacts with VDAC or the Bcl-2 family of pro-apoptotic members by immunoprecipitation, using an anti-His antibody or anti-MAV2054 antibody. However, MAV2054 showed no direct interaction with VDAC or Bcl-2 family members.

The induction of host cell apoptosis in response to a bacterial pathogen can be considered from two perspectives: as a virulence mechanism of the pathogen or as a defence mechanism of the host^55^. Most research indicates that the apoptosis of macrophages infected with mycobacteria functions as a host defence mechanism to eliminate the bacteria. However, the induction of apoptosis as a virulence strategy to establish and spread mycobacterial infection has been reported^56^,^57^,^58^. In this study, BMDMs infected with M. *smegmatis* expressing MAV2054 showed a significantly increased apoptotic response and more significant ROS production and ΔΨ_m_ collapse compared to in the mock strain. Surprisingly, the intracellular growth of *M. smegmatis* expressing MAV2054 within macrophages was significantly decreased. All mice infected with high doses of *M. smegmatis* died within 5 days, but mice with *M. smegmatis* expressing MAV2054 all survived. Collectively, our data suggest that the apoptosis of macrophages infected with *M. avium* represents a host defence mechanism against mycobacteria. To confirm this, however, precise molecular and cellular mechanisms by which MAV2054 decreases the intracellular survival rate of the bacteria via the mitochondrial apoptotic death pathway should be investigated.

In summary, the present study provides insights into the mechanisms by which MAV2054 induces apoptosis via the mitochondrial pathway in macrophages. Importantly, it increases the understanding of the pathogenic mechanism of *M. avium*, as indicated by the fact that MAV2054 expression in the bacteria suppressed its intracellular growth in macrophages and increased the survival rate of infected mice.

## Materials and Methods

### Animals

Specific pathogen-free, 5–6 week-old, female C57BL/6 (H-2K^b^ and I-A^b^) mice were purchased from the Jackson Laboratory (Bar Harbor, ME, USA), and were maintained under barrier conditions in a biohazard animal room at the Medical Research Center of Chungnam National University (Daejeon, Korea). The animals were fed sterile, commercial mouse diet and were provided water *ad libitum*. All animal experiments were approved by the Institutional Research and Ethics Committee at Chungnam National University (permission number: CNU-00517). All animal procedures were performed in accordance with the guidelines of the Korean Food and Drug Administration.

### Reagents and antibodies

Anti-PARP, anti-β-actin, anti-caspase-3, anti-caspase-9, VDAC, anti-phosphorylated ERK1/2, anti-phosphorylated p38, anti-phosphorylated JNK, anti-phosphorylated IκBα, anti-ERK1/2, anti-p38, anti-JNK, anti-Bcl-2, and anti-IκBα were purchased from Cell Signaling Technology, Inc. (Beverly, MA, USA). α-Tubulin, anti-Bax (6A7) and His-probe were obtained from Santa Cruz Biotechnology (Santa Cruz, CA, USA). Antibodies against cytochrome *c* (for immunofluorescence, clone 6H2.B4; for western blot analysis, clone 7H8.2C12) were acquired from BD Pharmingen (San Diego, CA, USA), and the anti-cytochrome oxidase subunit IV (COX IV) antibody was purchased from Abcam (Cambridge, UK). Dichlorodihydrofluorescein diacetate (H_2_DCFDA), MitoSOX, DAPI, and DiOC_6_ were obtained from Molecular Probes (Eugene, OR, USA) and z-VAD-fmk, *N*-acetyl-cysteine (NAC), horseradish peroxidase-conjugated anti-mouse IgG and anti-rabbit IgG, and specific inhibitors of ERK (U0126), p38 mitogen-activated protein kinase (MAPK; SB203580), JNK (SP600125), and nuclear factor-κB (NF-κB; BAY 11-7082) were purchased from Calbiochem (San Diego, CA, USA). Anti-F4/80 and anti-CD11b were performed as recommended by the manufacturer (eBioscience, San Diego, CA, USA).

### Recombinant MAV2054 protein, native Ag85 protein, and anti-MAV2054 antibody

Recombinant MAV2054 protein from *Escherichia coli* was produced and prepared as described previously^26^. Ag85 was purified from the culture filtrate protein of *M. tuberculosis* H37Rv as previously described by Lim *et al*.^28^. Prepared proteins were incubated overnight at 4 °C with polymyxin B-agarose beads (Sigma-Aldrich, St. Louis, MO, USA) to remove endotoxin. The endotoxin content of the final prepared proteins was very low (<0.05 U/mL). To obtain antiserum against MAV2054, BALB/c mice were immunised intraperitoneally with 25 μg of purified recombinant MAV2054 emulsified in incomplete Freund’s adjuvants. The mice were injected with the antigen three times at intervals of 2 weeks, and the serum was collected 1 week after the final immunisation.

### Cell culture

Murine macrophage cell line RAW264.7 cells were maintained in Dulbecco’s modified Eagle’s medium (DMEM; Lonza, Walkersville, MD, USA) supplemented with 10% foetal bovine serum (FBS; Hyclone, Logan, UT, USA), 1% HEPES, and 1% l-glutamine at 37 °C with 5% CO_2_. Bone-marrow-derived macrophages (BMDMs) were isolated from femurs and tibias of C57BL/6 mice (6–8-week old) and then differentiated by growth for 3–5 days in medium containing M-CSF (25 *μ*g/mL; R&D Systems, Minneapolis, MN, USA).

### ELISA for cytokines

The culture supernatants were collected from RAW264.7 cell and BMDMs stimulated with various concentrations of MAV2054, LPS, or Ag85. Sandwich ELISAs for detecting the cytokines and chemokines in the culture supernatants were performed as recommended by the manufacturer (eBioscience). Plates were read on a Vmax kinetic microplate reader (Molecular Devices Co., Sunnyvale, CA, USA) at 450 mm.

### MTT assay

RAW264.7 macrophages were seeded and stimulated with different concentrations of the recombinant protein in a final volume of 250 μL RPMI per well. The cell culture was incubated at 37 °C with 5% CO_2_. Cell cytotoxicity was tested by MTT assay by adding 10 μL of 5 mg/mL MTT. The cells were incubated at 37 °C for 3–4 h, and then, 100 μL of DMSO was added, and absorbance was measured at 570 nm.

### DNA fragmentation assay (apoptosis ELISA)

Cells were seeded in 96-well flat-bottom culture plates. After incubation with recombinant MAV2054 proteins, cells were collected, washed with phosphate-buffered saline (PBS), and processed for quantification of cytoplasmic histone-associated DNA fragments formed during apoptosis, using an enzyme-linked immunosorbent assay Cell Death Detection ELISA PLUS kit (Roche Diagnostic Corporation, Indianapolis, IN, USA).

### Annexin V/PI staining

To confirm the results of morphological analysis, Annexin V/propidium iodide (PI) staining was performed. For Annexin V/PI assays, cells were stained with Annexin V-FITC and PI and then monitored for apoptosis by flow cytometry in accordance with the manufacturer’s protocol (BD Biosciences, Franklin Lakes, NJ, USA). Briefly, 0.5 × 10^6^ cells were washed twice with PBS and stained with 5 μL of Annexin V-FITC and 10 μL of PI (5 μg/mL) in 1 × binding buffer (10 mM HEPES, pH 7.4, 140 mM NaOH, 2.5 mM CaCl_2_) for 15 min in the dark. Apoptotic cells were quantified using the FACS Canto II cytometer (BD Biosciences). Both early apoptotic (Annexin V-positive, PI-negative) and late apoptotic (double-positive for Annexin V and PI) cells were detected. Data were processed using Flow Jo (Tree Star, Ashland, OR, USA).

### Subcellular fractionation

Cells were harvested with PBS. The cytosolic and mitochondrial fractions were purified using the Subcellular Protein Fraction Kit for Cultured Cells (Thermo Scientific, Waltham, MA, USA).

### Immunoblotting

The cells were harvested at the indicated time points and then incubated in lysis buffer (10 mm Tris-HCl, pH 7.4, 5 mm EDTA, 150 mM NaCl, 1% Triton X-100, 1 mM PMSF, and protease inhibitor cocktail) for 30 min on ice. Protein concentrations were determined by the Bradford assay, and equal amounts of proteins were separated by SDS–PAGE, electroblotted onto polyvinylidine difluoride membranes, blocked, and incubated overnight with primary antibodies. The blots were probed with primary antibodies at optimised concentrations followed by incubation with horseradish peroxidase-conjugated secondary antibodies. The enhanced chemiluminescence system (ECL; Millipore Corporation, Billerica, MA, USA) was used, followed by exposure to chemiluminescence film to visualize proteins.

### Subcellular fractionation of *M. avium*

Generally, the *M. avium* was grown to an OD_600_ ~0.8. Next, the cells were harvested, resuspended in 10 mL lysis buffer (PBS, 1 mM PMSF, protein cocktail inhibitor) and lysed by sonication. The lysates were centrifuged at 3,000 *g* for 30 min to separate whole cell lysates (WCL) from the supernatant. WCL was ultra-centrifuged at 27,000 *g* for 30 min to obtain the cell wall pellet (CW). The supernatant from the WCL fraction was further ultra-centrifuged at 100,000 *g* for two hours to separate the cell membrane fraction and obtain the pellet fraction(CM) and supernatant (cytoplasmic, Cyto). The CW and CM fraction were washed once with lysis buffer, re-centrifuged, and subsequently, re-suspended in 0.5 mL of lysis buffer. All centrifugation steps were performed at 4 °C.

### Measurement of ROS

Intracellular ROS were evaluated by staining cells with H_2_DCFDA (to measure total cellular H_2_O_2_) and/or MitoSOX (to measure the mitochondrial ROS superoxide). Cells were harvested with PBS. For ROS staining, 10 μM H_2_DCFDA and/or MitoSOX were added to cells for 30 min at 37 °C in the dark, and then the cells were washed with PBS. Samples were immediately analysed on a FACS Canto II cytometer (BD Biosciences) and the data were processed using Flow Jo software.

### Assessment of mitochondrial membrane potential (ΔΨ_m_)

Mitochondrial membrane potential was assessed by measuring the retention of the lipophilic cationic dye DiOC_6_ in the mitochondria. Cells were harvested and incubated in DiOC_6_ solution (10 nM in fresh medium) for 30 min at 37 °C in the dark. The cells were then washed and resuspended in PBS. Immediately after PBS washing, ΔΨ_m_ was measured by sorting the cells using the FACS Canto II (BD Biosciences). Dead cells were excluded by forward and side-scatter gating. Data were analysed using Flow Jo software.

### Confocal microscopy

Cells were seeded onto glass coverslips in 12-well plates. The subcellular localization of MAV2054 was analysed using a confocal microscope (LSM510 META; Carl Zeiss, Heidelberg, Germany). The cells were incubated with Mitotracker Red (Molecular Probes). After washing, the cells were fixed with 4% paraformaldehyde for 20 min, permeabilised with 0.1% Triton X-100 in PBS for 10 min, blocked with PBS containing 3% bovine serum albumin for 1 h, stained with primary anti-MAV2054 antibody for 1 h, and then stained with secondary antibody (anti-mouse IgG FITC; DAKO, Glostrup, Denmark) for 1 h. DAPI staining was conducted for 10 min. Cells were washed with PBS between each step. The cytosol localization of MAV2054 was analysed using a confocal microscope. The cells were fixed, permeabilised, and stained with an anti-MAV2054 antibody followed by an anti-mouse IgG FITC (DAKO). After DAPI staining with Texas Red®-X phalloidin (Molecular Probes) for 20 min, the cells were imaged under a confocal microscope (Carl Zeiss).

### Transformation and isolation of recombinant *M. smegmatis* strains

Preparation of *M. smegmatis* competent cells and electroporation procedures were performed following standard procedures^59^. Briefly, *M. smegmatis* stain mc^2^155 was grown in 7H9/ADC/Tw (7H9 containing 10% ADC (Microbiol, Cagliari, Italy) and 0.05% Tween 80 (Sigma)) until mid-log phase. Cells were then harvested and washed twice with cold 10% glycerol and resuspended in the same buffer. Next, 0.2 mL of competent cells was electroporated using standard settings: 1.5 kV, 1000 Ω, and 25 μF, using the Cene Pulser apparatus (Bio-Rad, Hercules, CA, USA). The cells were harvested with 1 mL of warm 7H9/ADC/Tw, incubated for 4 h at 37 °C with gentle shaking, and differential dilutions of the culture were plated into 7H11/OADC plates containing 50 *μ*g/mL hygromycin (Sigma). Plates were incubated at 37 °C for 3–4 days to obtain the recombinant strains.

### Intracellular survival assay

BMDMs were infected with *M. smegmatis* at a multiplicity of infection (MOI) of 5 for 2 h at 37 °C, 5% CO_2_. Amikacin (200 μg/mL; Sigma) was added to each well and incubated for 2 h. After incubation, the supernatant was collected and the cells were lysed with sterile distilled water for 20 min. Cell lysates and collected supernatants were serially diluted and plated onto 7H10 agar plates to determine the “input” bacterial numbers.

### Mouse infection and lung cell preparation

The mice C57BL/6 were divided into two groups: *M. smegmatis*, and *M. smegmatis* expressing MAV2054, with each group containing five mice. Mice in the two groups were inoculated intravenously with 1 × 10^7^ CFU of either *M. smegmatis* or *M. smegmatis* expressing MAV2054, diluted with 200 μL of PBS. Mouse lungs were cut into pieces and agitated in 5 mL of cellular dissociation buffer (RPMI medium containing 0.1% collagenase type IV (Worthington Biochemical Corporation, Lakewood, NJ, USA), 1 mM CaCl_2_, and 1 mM MgCl_2_) for 15 min at 37 °C. Then lungs were homogenized and aggregates were filtered through a 40-μm cell strainer. Alveolar macrophages were analysed by flow cytometry using CD11b^+^ and F4/80^+^, and Annexin V/PI staining.

### Statistical analysis

All experiments were performed independently and repeated at least three times. A *P-*value < 0.05 was considered to be statistically significant in Student’s *t*-tests. Data are presented as the mean ± 95% confidence interval.

## Additional Information

**How to cite this article**: Lee, K.-I. *et al*. *Mycobacterium avium* MAV2054 protein induces macrophage apoptosis by targeting mitochondria and reduces intracellular bacterial growth. *Sci. Rep.* **6**, 37804; doi: 10.1038/srep37804 (2016).

**Publisher's note:** Springer Nature remains neutral with regard to jurisdictional claims in published maps and institutional affiliations.

## Supplementary Material

Supplementary Information
