# Supplementary material for: Mycobacterium avium MAV2054 protein induces macrophage apoptosis by targeting mitochondria and reduces intracellular bacterial growth
Source: Sci Rep. 2016 Nov 30;6:37804. doi: 10.1038/srep37804 (PMC5129020; doi:10.1038/srep37804)
Supplement: Supplementary Information [file srep37804-s1.pdf]

Supplementary Infomation

Mycobacterium avium MAV2054 protein induces macrophage apoptosis through targeting to mitochondria and reduces intracellular growth of the bacteria.

Kang-In Lee[1,2], Jake Whang[1,2], Han-Gyu Choi[1,2], Yeo-Jin Son[1], Haet Sal Jeon[1,2], Yong Woo Back[1,2], Hye-Soo Park[1,2], Seungwha Paik[1,2], Jeong-Kyu Park[1,2], Chul Hee Choi[1,2,*], Hwa-Jung Kim[1,2,*]

[1] Department of Microbiology, and [2] Department of Medical Science, College of Medicine, Chungnam National University, Daeieon 301-747, Republic of Korea

*Corresponding authors

Hwa Jung Kim: Tel) +82-42-580-8242, Fax)+82-42-585-3686, email) hjukim@cnu.ac.kr

Chul Hee Choi: Tel)+82-42-580-8246, Fax)+82-42-585-3686, email) choich@cnu.ac.kr

Supplementary figure 1

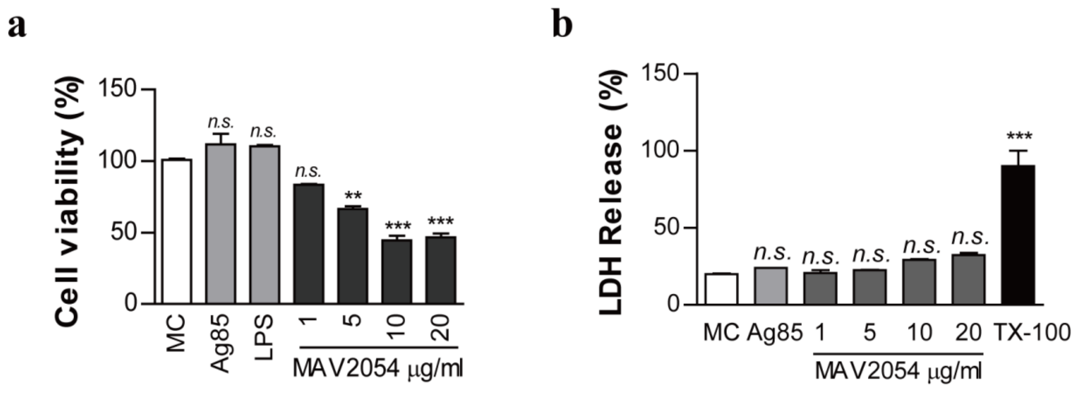

Supplementary figure 1: Determination of LDH release in RAW264.7 cells treated with MAV2054.
(a) Cell viability of RAW264.7 cells incubated with MAV2054 (1, 5, 10, and 20 μg/mL) for 24 h was determined by MTT assay. **(b)** RAW264.7 cells were incubated with the indicated concentrations of MAV2054 for 48 h. LDH release was measured by Cytotoxicity Detection Kit. Positive control was generated by treating cells with 1% Triton X-100 (TX-100) for 1 h prior to the onset of the assay. All data shown are the mean values ± SD of at least two experiments. **P < 0.01, and ***P < 0.001 cell treated with media control (MC) versus treated cells with antigens.

Supplementary figure 2

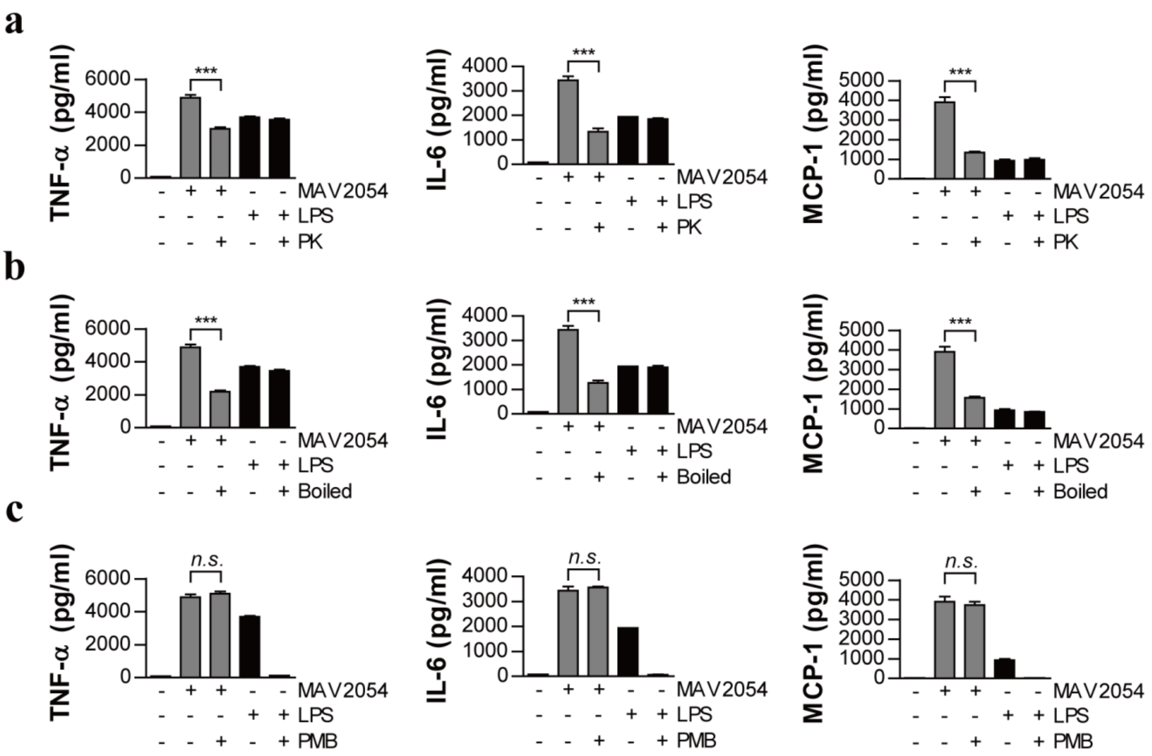

Supplementary figure 2: Confirmation of endotoxin decontamination from purified MAV2054.

MAV2054 (10 μg/mL) was **(a)** Denatured by heating for 1 h at 100°C. **(b)** Digested with proteinase K (10 μg/mL) for 1 h at 37°C. **(c)** Pre-treated with polymyxin B (50 μg/mL) for 1 h prior to stimulation of macrophages. After 24 h, the quantities of TNF-α IL-6, and MCP-1 in the culture medium were measured by ELISA. All data expressed as the mean values ± SD (n = 3 samples) are shown. One representative plot out of two independent experiments is shown; ***P < 0.001 compared to MAV2054-treated macrophages.

Supplementary figure 3

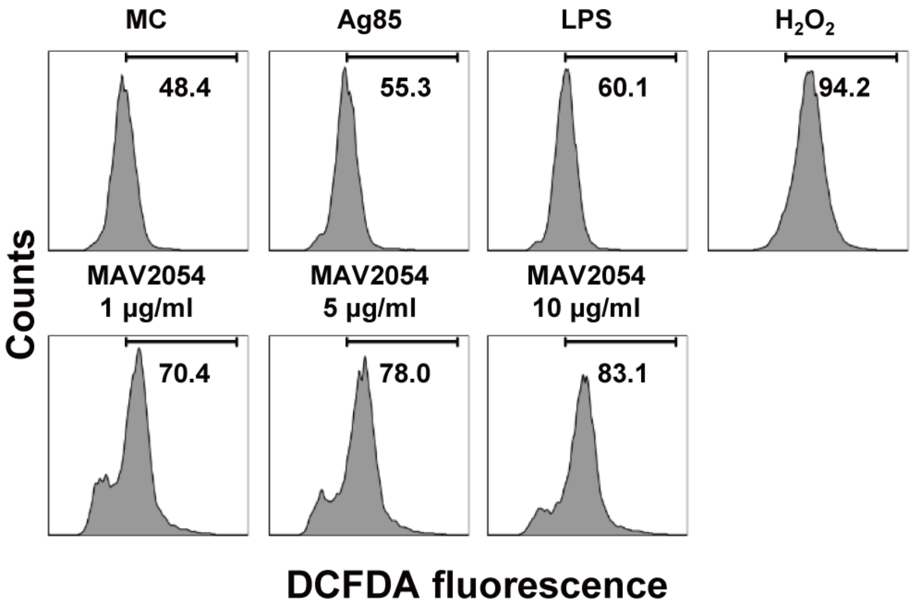

Supplementary figure 3: ROS production in MAV2054-treated macrophages was increased in a dose-dependent manner. RAW264.7 cells were treated with Ag85 (10 µg/mL), LPS (100 ng/mL), MAV2054 (1, 5, 10 µg/mL), and H_2O_2 (500 nM) for 24 h. The number indicates increased DCFDA fluorescence.

Supplementary figure 4

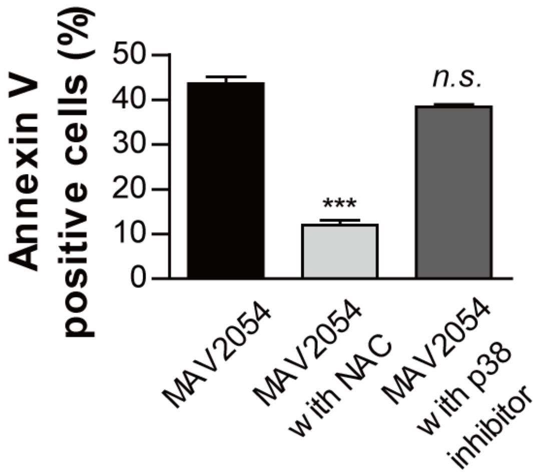

Supplementary figure 4: MAV2054-induced apoptosis was not inhibited by p38 inhibitor.

RAW264.7 cells were incubated with 10 µM of a p38 inhibitor (SB203580) and 10 mM of the ROS inhibitor NAC for 1 h prior to treatment with MAV2054 (10 µg/mL). After 24 h, apoptosis was analysed by flow cytometry using Annexin V/PI staining. Flow cytometric histograms are representative mean ± SD of three independent experiments. ***P < 0.001 for RAW264.7 cells incubated with MAV2054 *versus* those incubated with MAV2054+NAC or MAV2054+p38 inhibitor.

Supplementary figure 5

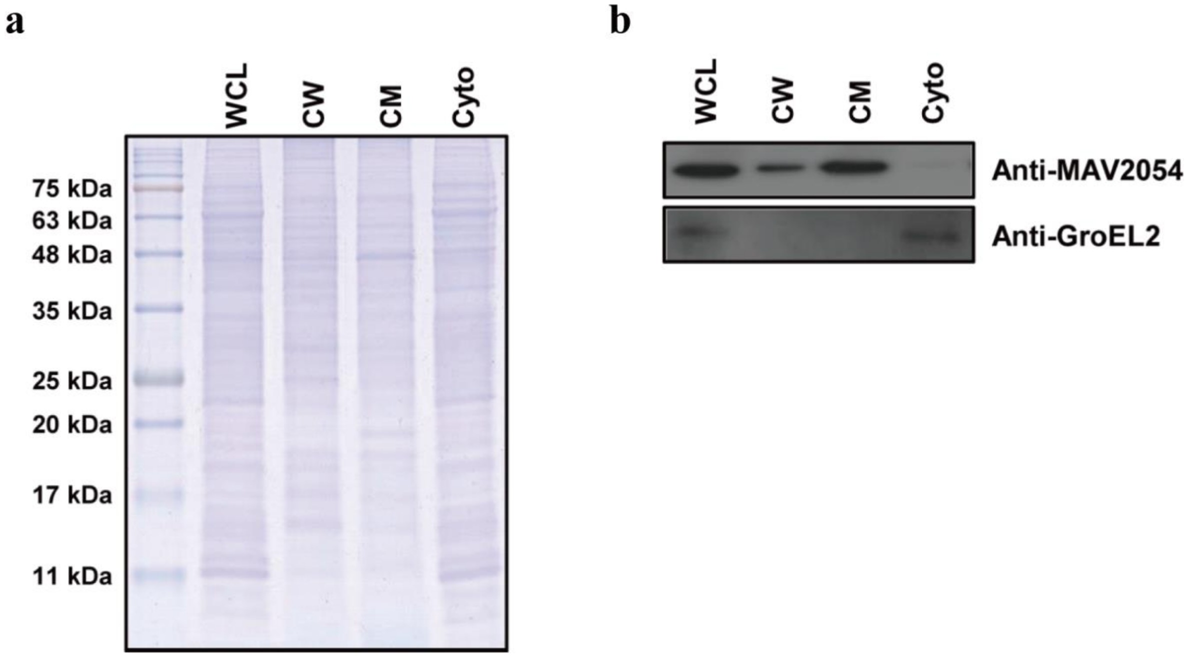

Supplementary figure 5: MAV2054 locates in cell membrane and wall fractions of *M.avium*. (a) Whole cell lysate (WCL) of *M.avium* was fractionated into cell wall (CW), cell membrane (CM), cytoplasmic fraction (Cyto) and each fraction (20 µg) was separated by SDS-PAGE and stained with Coomassie blue, and **(b)** analyzed by immunoblot using anti-MAV2054 antibody and anti-GroEL antibody. GroEL2 was detected as a cytoplasmic protein control.

Supplementary figure 6

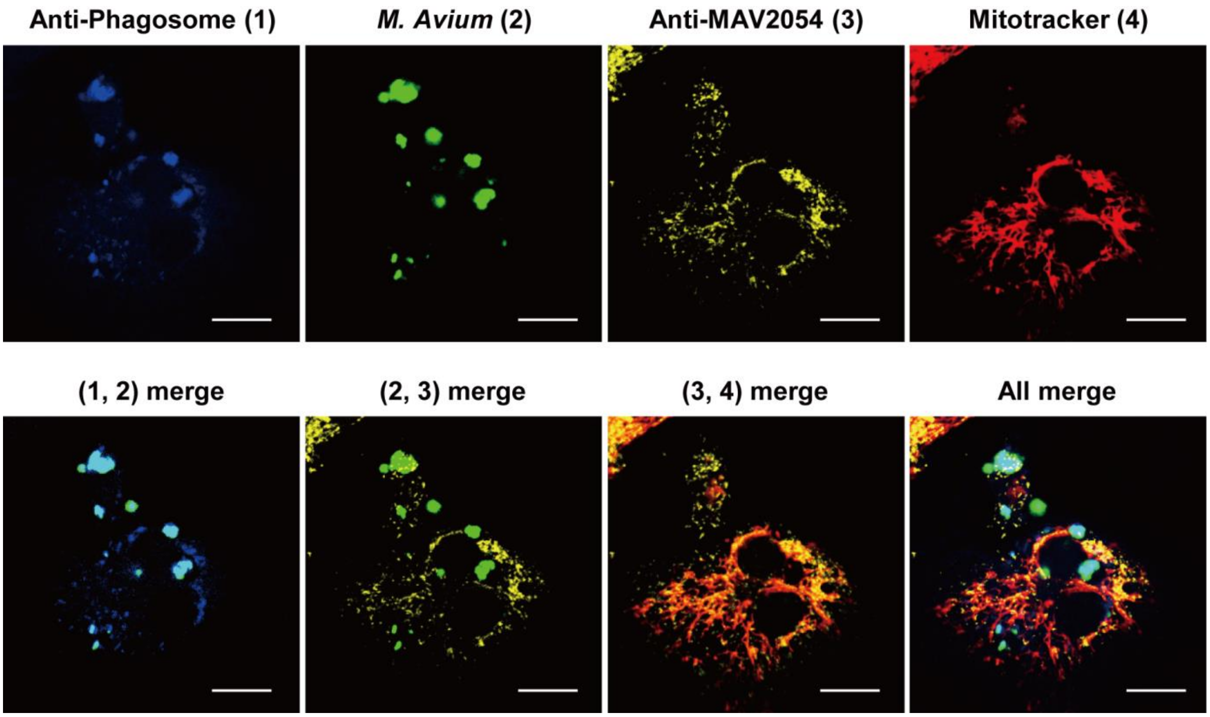

Supplementary figure 6: The *M.avium* infected macrophages secrete MAV2054 into cytoplasm and mitochondria. BMDMs were infected with *M. avium* at MOI of 10 for 20 h. Represent confocal images of *M. avium* infected cells. Phagosome (Rab7), *M.avium*, MAV2054, and Mitochondria were stained with alexa 350 (blue), CFSE (green), alexa 568 (yellow), and Mitotracker (red) respectively. Lower panel indicates co-localization merged images. Scale bar, 10 μm.

Supplementary figure 7

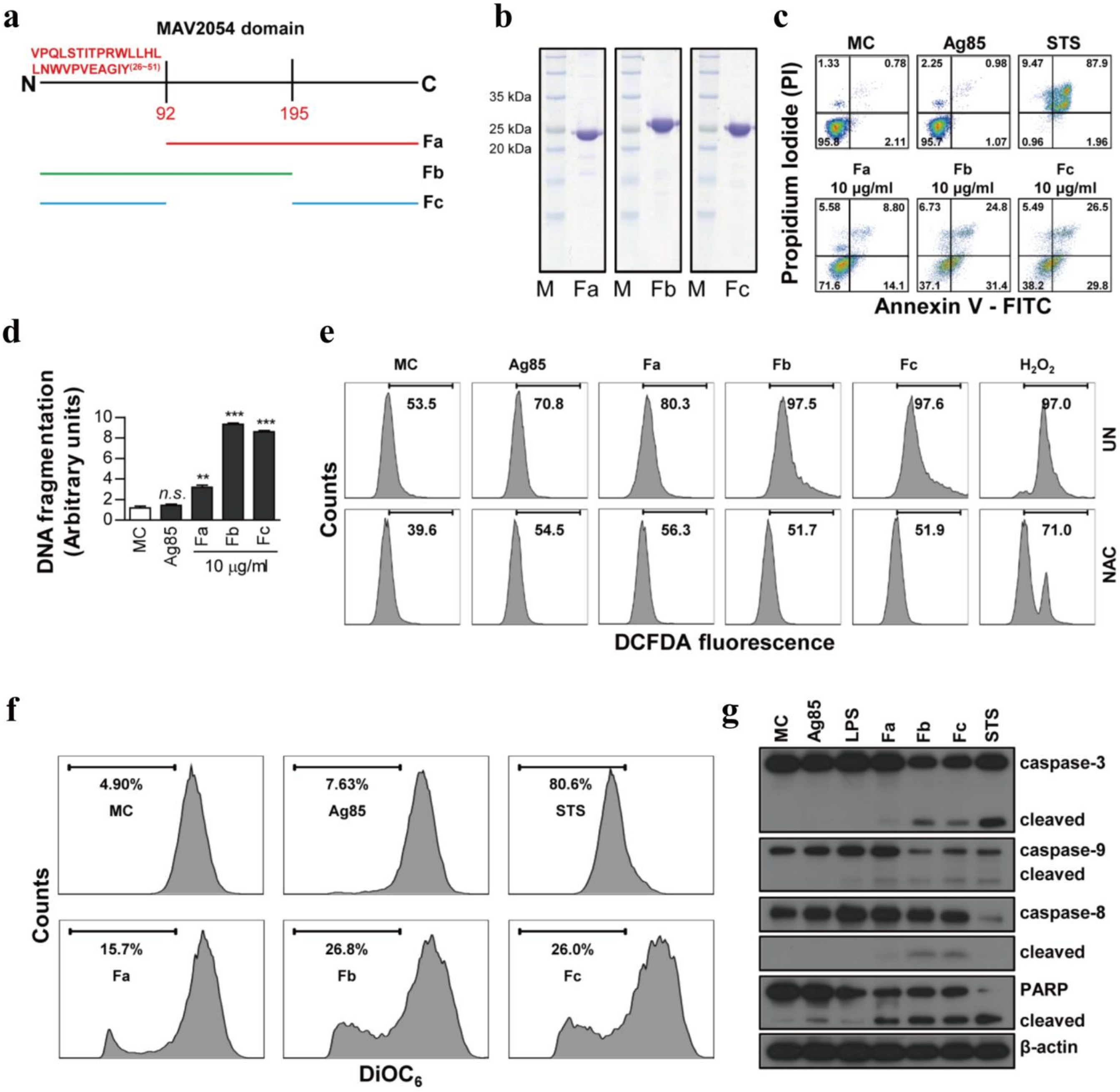

Supplementary figure 7. Mitochondrial target sequences located in N-terminal are essential for MAV2054-mediated apoptosis. (a) Three truncated forms of MAV2054 with or without the mitochondrial target sequences were constructed, and expressed in E.coli, and purified by Ni-NTA affinity chromatography **(b)** Fragment a (Fa) was a two-third of C-terminal, fragment b (Fb) was a two-third of N-terminal, and fragment c (Fc) was fusion fragment of C-terminal one-third and N-terminal one-third. Apoptosis of RAW264.7 cells treated with Ag85 (10 µg/mL), Fa (10 µg/mL), Fb (10 µg/mL), Fc (10 µg/mL), and STS (50 nM) for 24 h was measured by flow cytometry using Annexin V/PI staining **(c)** and by Cell Death Detection ELISA **(d)** $^{**}P < 0.01$, and $^{***}P < 0.001$ for antigen treatments compared to untreated medium control (MC). ROS levels **(e)** $\Delta\Psi_m$ **(f)** and caspase activity **(g)** in cells treated with each fragment were measured by flow cytometry or immunoblot.

Supplementary figure 8

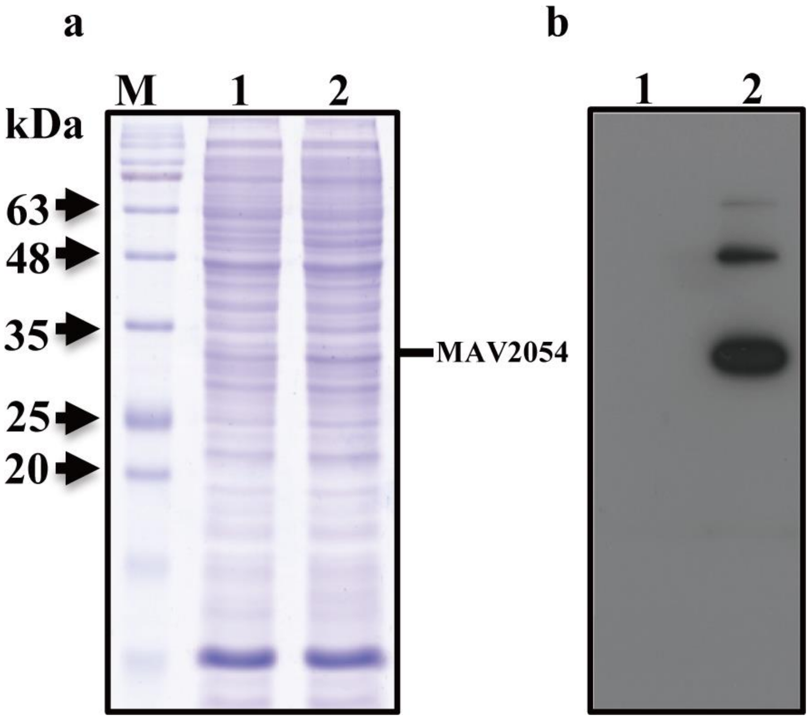

Supplementary figure 8: Comparison of the protein levels in the total extracts between *M. smegmatis* mock strain and MAV2054 expressing strain. Total sonic extracts from the both strain were subjected to SDS-PAGE and then analyzed by Coomassie blue stain (a) and Western blot (b) using anti-MAV2054 antibody (1:10,000). Lane 1, *M. smegmatis* mock strain; lane 2, *M. smegmatis* expressing MAV2054.
